# Optimization of Fermentation Medium for Extracellular Lipase Production from *Aspergillus niger* Using Response Surface Methodology

**DOI:** 10.1155/2015/497462

**Published:** 2015-08-20

**Authors:** Jia Jia, Xiaofeng Yang, Zhiliang Wu, Qian Zhang, Zhi Lin, Hongtao Guo, Carol Sze Ki Lin, Jianying Wang, Yunshan Wang

**Affiliations:** ^1^Shenzhen Leveking Bio-Engineering Co., Ltd., Guangdong, Shenzhen 518055, China; ^2^School of Energy and Environment, City University of Hong Kong, Tat Chee Avenue, Kowloon, Hong Kong; ^3^Shenzhen Institute of Technology, Guangdong, Shenzhen 518116, China

## Abstract

Lipase produced by *Aspergillus niger* is widely used in various industries. In this study, extracellular lipase production from an industrial producing strain of *A. niger* was improved by medium optimization. The secondary carbon source, nitrogen source, and lipid were found to be the three most influential factors for lipase production by single-factor experiments. According to the statistical approach, the optimum values of three most influential parameters were determined: 10.5 g/L corn starch, 35.4 g/L soybean meal, and 10.9 g/L soybean oil. Using this optimum medium, the best lipase activity was obtained at 2,171 U/mL, which was 16.4% higher than using the initial medium. All these results confirmed the validity of the model. Furthermore, results of the Box-Behnken Design and quadratic models analysis indicated that the carbon to nitrogen (C/N) ratio significantly influenced the enzyme production, which also suggested that more attention should be paid to the C/N ratio for the optimization of enzyme production.

## 1. Introduction

Lipases (triacylglycerol ester hydrolase EC 3.1.1.3) catalyze the hydrolysis of triglycerides into fatty acids and glycerol [1, 2] and under certain conditions can also catalyze the synthesis of esters through transesterification, thioesterification, and aminolysis [3–5]. Lipases exist widely in nature environment, especially in bacteria, yeasts, and filamentous fungi. In recent years, the interest in microbial lipases production has been increasing, because of their large potential in industrial applications for the synthesis of biopolymers and biodiesel, the production of enantiopure pharmaceuticals, agrochemicals, and flavour compounds [2, 6]. Both high-level production and the safety of the process and products are required to broaden the industrial application of lipases [6].

*Aspergillus niger* is one of the most important industrial microbes, which can produce more than 30 species of enzymes such as lipase [7–11], amylase [12, 13], cellulase [14, 15], pectinase [16], and glucose oxidase [17, 18]. It has been widely used to produce extracellular enzymes and organic acid for decades [19, 20]. The microorganism is Generally Recognized as Safe (GRAS) by the United States Food and Drug Administration [19]. Because of its excellent protein secretion capability, mature fermentation, and posttreatment process and safety,* A. niger* becomes one of the most important species in industrial application [21].* A. niger* enzymes such as lipase could be utilized as auxiliary material or additive in food, medicine, and feed industry [19]. Maximization of the lipase yield is a prerequisite for the immense potential of* A. niger* lipase. The composition of fermentation medium has significant effect on microorganism metabolism [22], which is desired to optimize for the maximum yields of lipase.

Response Surface Methodology (RSM) is a mathematical and statistical method that can overcome some drawbacks like time consumption and high cost. It is widely used to solve multiple variable problems in different biotechnological processes [13, 23–27]. RSM has been successfully applied to evaluate and optimize the effect of process parameters in the production of lipase. Hatzinikolaou et al. [24] used RSM to study the effect of carbon and nitrogen sources on the extracellular lipase production from* A. niger*. A maximum lipase activity of 42.4 U/mL was obtained in the optimum medium with a combination of corn oil and peptone. Also using RSM, Kaushik et al. [23] reported that sunflower oil, glucose, peptone, agitation rate, and incubation temperature were the most influential parameters in the production of extracellular lipase from* A. carneus*. A 1.8-fold increase in production, with the final yield of 12.7 IU/mL, was obtained under the optimum medium and operation. Box-Behnken Design (BBD) is a RSM suitable for fermentation optimization in shake flasks, in which the most influential parameters can be determined by the minimum numbers of experiments [25]. In this study, we aimed to improve the yield of lipase production through optimization of the fermentation medium composition for* A. niger* G783, which is an industrial production strain. In this paper, the influential parameters were determined and optimized by a series of single-factor experiments and BBD method, respectively, which can also evaluate the effect and relationship of different medium components.

## 2. Materials and Methods

### 2.1. Strains and Chemicals

*A. niger* G783, a genetic stability lipase high-yielding industrial production strain, was genetically engineered by physical and chemical mutagenesis from* A. niger* CICC 2475 (China Center of Industrial Culture Collection), which was preserved in Shenzhen Leveking Bio-Engineering Co., Ltd.

Glucose, sucrose, corn starch, dextrin, soybean meal, peptone, beef extract, yeast extract, salt, and agar were obtained at analytical grade from chemical suppliers in China. Olive oil and soybean oil were purchased from local markets (Shenzhen, China).

### 2.2. Medium

Yeast extract peptone dextrose (YPD) medium contained (per liter) 10 g yeast extract, 20 g peptone, 20 g glucose, 15.0 g agar, and pH 7.0.

Seed medium (SM) contained (per liter) 30.0 g glucose, 3.0 g NaNO_3_, 0.5 g NaH_2_PO_4_, 0.1 g K_2_SO_4_, and pH 7.2.

Fermentation medium 1 (FM1, per liter) contained 20.0 g glucose, 10.0 g corn starch, 40.0 g soybean meal, 10.0 g olive oil, 10.0 g NaNO_3_, 2.0 g NaH_2_PO_4_, 0.2 g K_2_SO_4_, 5.0 g CaCO_3_, and pH 7.2.

### 2.3. Strain Cultivation

The inoculum was produced in YPD medium in slant. Shake flasks of 250 mL containing 25 mL of seed medium were incubated in a shaker (220 rpm) at 30°C, for 20–32 h. Lipase production was carried out with a 2% (v/v) inoculum of* A. niger* G783 in 500 mL shaken flasks with 50 mL of FM1 and incubated at 30°C in a shaker (220 rpm) for about 48 h. The broth was refrigerated at 4°C and centrifuged at 10,000 rpm for 10 minutes, and the supernatant was obtained as crude enzyme solution that was used for lipase activity analysis.

### 2.4. Single-Factor Experiment

To determine the influential parameters on the lipase production, we carried out a single-factor experiment. The value of the factors was set based on FM1 medium. The medium was divided into five components, primary carbon sources, secondary carbon sources, nitrogen sources, oils, and inorganic salts. The composition of components and the experimental sequence for the single-factor experiment are shown in Table 1. Fermentation was performed in 500 mL shake flasks with 50 mL medium and incubated at 30°C in a shaker (220 rpm) for about 48 h. The broth was refrigerated at 4°C and then centrifuged at 10,000 rpm for 10 minutes. The supernatant was obtained as crude enzyme solution that was used for lipase activity analysis. All components were analyzed independently, and every test was performed in triplicate. The influential factors and levels for the enzyme activity were evaluated.

### 2.5. BBD Design

Box-Behnken Design (BBD), one of RSM design, with a three-level factorial design was used as the experimental design model to optimize the influential parameters for enhancing lipase production. According to the results of single-factor experiment, the levels of the variables and the experimental design (according to Design-Expert 8.0) are shown in Table 2. The lipase activities in volume were associated with simultaneous changes in corn starch concentration (8, 10, and 12 g/L), the soybean meal concentration (30, 35, and 40 g/L), and the soybean oil concentration (5, 10, and 15 g/L) of fermentation medium. In this study, 17 experiments planned with the BBD design were carried out for building quadratic models, with five replications of the center points to estimate the experimental errors accordingly. The corresponding fermentation was performed in 500 mL shake flasks with 50 mL medium and incubated at 30°C in a shaker (220 rpm) for about 48 h. The broth was refrigerated at 4°C and centrifuged at 10,000 rpm for 10 minutes, and the supernatant was obtained as crude enzyme solution that was used for lipase activity analysis.

A software named “Design-Expert 8.0” was used to analyze the experimental results, build the regression model, and predict the optimal processing parameters [28].

### 2.6. Lipase Activity Assay

Lipase activity in the fermentation broths was analyzed according to a slightly modified NaOH titration method [29]. The assay mixture contained 5 mL of the olive oil emulsion, 4 mL of 50 mmol/L glycine-NaOH buffer (pH 9.4), and 1 mL of enzyme solution. The reaction was preceded at 36°C for 15 minutes and stopped by adding 20 mL of 95% ethanol and 10 mL 30% NaCl. One unit of the lipase activity was defined as the amount of enzyme required to release 1 *μ*mol of fatty acid per minute under assay condition [30].

## 3. Result and Discussion

### 3.1. Single-Factor Optimization of Fermentation Media

According to our previous works and the characteristics of* A. niger* G783, the levels of the variables were set to be close to the central point in the single-factor experiments. As shown in Table 1, the levels of the variables were designed and the single-factor experiments were performed as the following sequence: primary carbon source, secondary carbon source, nitrogen source, oil, and inorganic salt. The optimum variable and level that were determined in every step would be used in the next steps.

**Figure 1:**
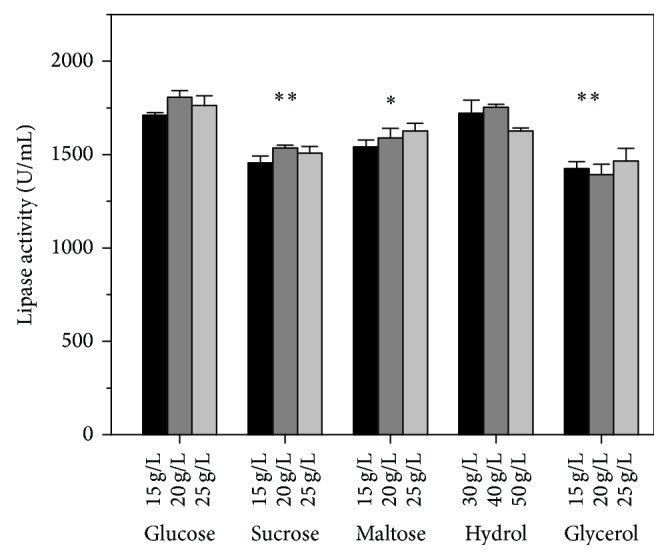
The effect of primary carbon sources on lipase activity obtained by* A. niger* G783. The medium was modified based on FM1. The values are average of three independent experiments and the error bars represent standard deviation.

**Figure 2:**
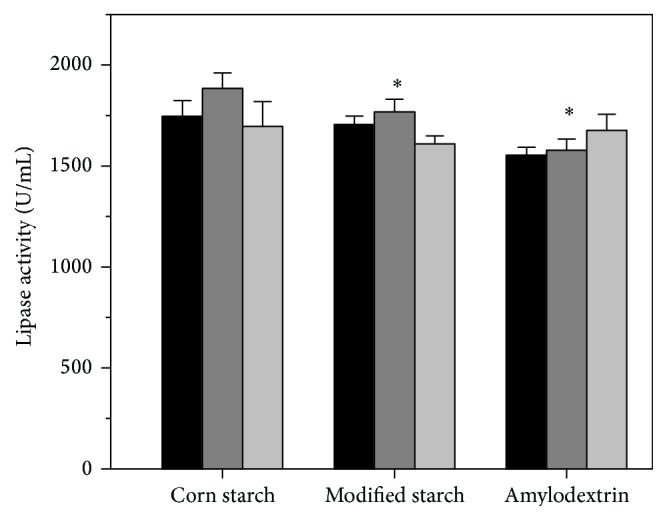
The effect of secondary carbon sources on lipase activity obtained by* A. niger* G783. The medium was modified based on FM1. The values are average of three independent experiments and the error bars represent standard deviation.

As carbon catabolite repression (CCR) existed in* Aspergillus* sp. [31, 32], two types of carbon source, primary and secondary carbon sources, are thought to play different roles in the metabolism of* Aspergillus* sp. Therefore, these carbon sources were analyzed independently. Glucose, maltose, corn starch hydrolyzate (hydrol), sucrose, and glycerol were selected as the primary carbon source, while corn starch, modified starch, and dextrin were selected as secondary carbon source (Table 1). As shown in Figure 1, higher lipase activity was obtained when glucose was used as primary carbon source. Using glucose as the primary carbon source was significantly higher than using maltose, sucrose, or glycerol. However, the lipase activity obtained from hydrol was not significantly different than using glucose, because the major content of hydrol was glucose. This result suggested that glucose was the best primary carbon source for lipase production. Moreover, the lipase production was similar to each other between 15 and 25 g/L glucose. To make sure the carbon source is enough, 25 g/L glucose was used as the primary carbon source in the following studies. For the three types of secondary carbon source, corn starch was the best secondary carbon source on the lipase production (Figure 2). Among the concentration range of 5–15 g/L corn starch, the best condition was 10 g/L corn starch. Thus, the amounts of carbon sources were designed as 25 g/L glucose and 10 g/L corn starch in FM1.

**Figure 3:**
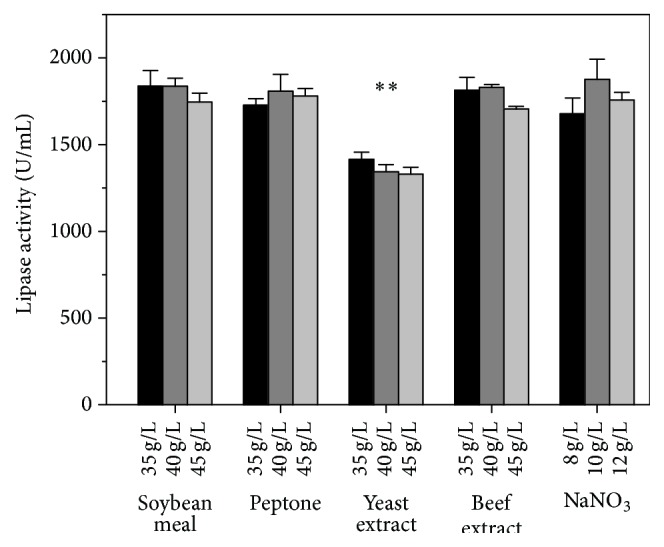
The effect of nitrogen source on lipase activity obtained by* A. niger* G783. The medium was modified based on FM1. For the study on the effect of NaNO_3_, 40 g/L soybean meal was supplemented in all runs. The values are average of three independent experiments and the error bars represent standard deviation.

During fermentation, the availability of precursors for protein synthesis [33] and the nitrogen source [34] are both important in the production of extracellular enzymes. Nitrogen source would also significantly affect the pH of the medium. To obtain an insight for the effect of different nitrogen sources, various inorganic and organic nitrogen sources were investigated in lipase production. As shown in Figure 3, the activity of lipase has no significant difference when using peptone, beef extract, and soybean meal with or without NaNO_3_, respectively. However, lipase activity decreased significantly when yeast extract was used as nitrogen source. Thus, soybean meal was selected as nitrogen source because of its low cost and the ease of accessibility. Since the soybean meal was assumed to contain most of the necessary nutrients that lipase production needs, no other nitrogen supplements were necessary [26]. Herein, the lipase activity was not significantly different when supplemented with sodium nitrate, which suggested that sodium nitrate can be omitted.

**Figure 4:**
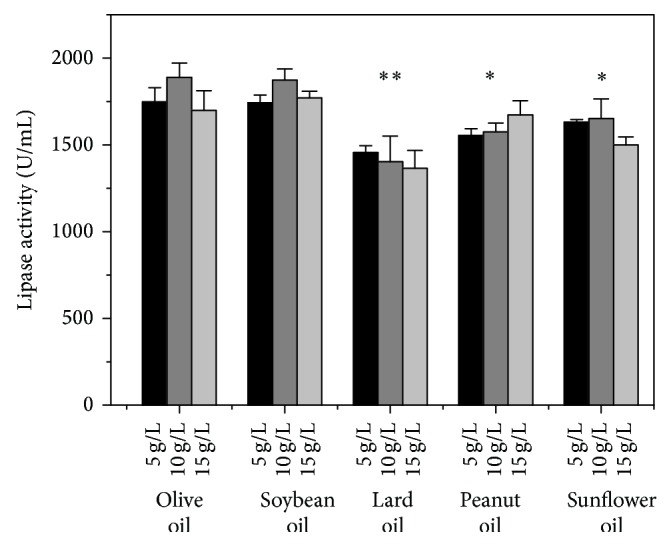
The effect of the oil on lipase activity obtained by* A. niger* G783. The medium was modified based on FM1. The values are average of three independent experiments and the error bars represent standard deviation.

**Figure 5:**
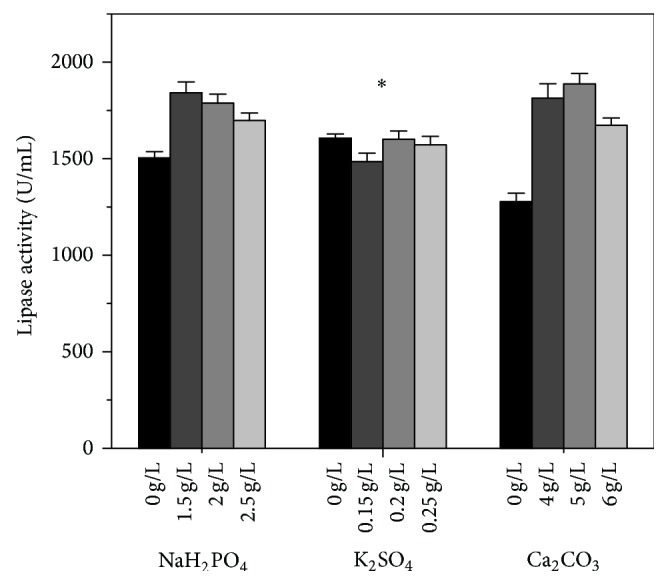
The effect of the inorganic salt on lipase activity obtained by* A. niger* G783. The medium was modified based on FM1. The values are average of three independent experiments and the error bars represent standard deviation.

Oils could be served as carbon source and inducer of lipase synthesis during* A. niger* fermentation [35].  Figure 4 showed that olive oil and soybean oil were both outstanding for lipase production, compared to lard oil, peanut oil, and sunflower oil. As it is of low cost and easily acquired, soybean oil was selected to replace olive oil. Although the effect of sodium phosphate monobasic and calcium carbonate on the lipase production was greater than potassium sulfate (Figure 5), all inorganic salts chosen in the single-factor experiments are essential for the basic metabolism of* A. niger*. The results indicated that the concentration of inorganic salts should be 1.5 g/L NaH_2_PO_4_, 0.2 g/L K_2_SO_4_, and 5 g/L CaCO_3_, respectively. Based on the results of single-factor experiments, a new fermentation medium (FM2) was established as follows (per liter): 25 g glucose, 10 g corn starch, 40 g soybean meal, 10 g soybean oil, 12 g NaNO_3_, 1.5 g NaH_2_PO_4_, 0.2 g K_2_SO_4_, 5 g CaCO_3_, and pH 7.2.

### 3.2. BBD Model Fitting and Data Analysis

According to the single-factor experiments, three influential factors (*A*: corn starch, *B*: soybean meal, and *C*: soybean oil) in FM2 for G783 fermentation were selected for BBD design and quadratic models analysis (Table 2). Parameters of the BBD design were set as follows: factor = 3, liver = 3, and runs = 17 (including 5 replications of the center points), and lipase activity was set as response value (*Y*). After BBD, the result was then analyzed by standard analysis variance (ANOVA) according to the simulated quadratic equation:

(1) Ycoded=2071.8+97.00A+114.25B+76.50C−103.75AB−43.25AC+16.25BC−A2−227.03B2−76.02C2.

The variance analysis results were listed in Table 3. This model processes high reliability, high fitting degree, and deviation with coefficient of determination *R* ^2^ = 0.9826 and adequate precision of 22.523. Furthermore, the *P* value of this model was significant (*P* < 0.01) and the lack of fit was not significant (*P* = 0.9057 > 0.05), which indicated that the residual might be caused by random error and this model is adequate. On the whole, this model could be used for evaluation, optimization, and prediction of lipase fermentation process. The variance analysis of three factors (*A*, *B*, and *C*) showed that *A*, *B*, *C*, *A* ^2^, *B* ^2^, and *C* ^2^ have significant effect on enzyme production (*P* < 0.05) as listed in Table 3. This model predicted that these three factors were significant affecting the lipase production. In the medium, corn starch, soybean meal, and soybean oil are the major source for supply carbon or nitrogen resource to maintain the normal microbe metabolism and protein synthesis. Additionally, various amounts of these three factors also affect the carbon to nitrogen ratio (C/N), which could affect the metabolic pathway of the microorganism. We discussed about the effect of C/N ratio thereinafter. Therefore, the concentrations of corn starch, soybean meal, and soy oil bean in the medium have significant effect on enzyme production.

**Figure 6:**
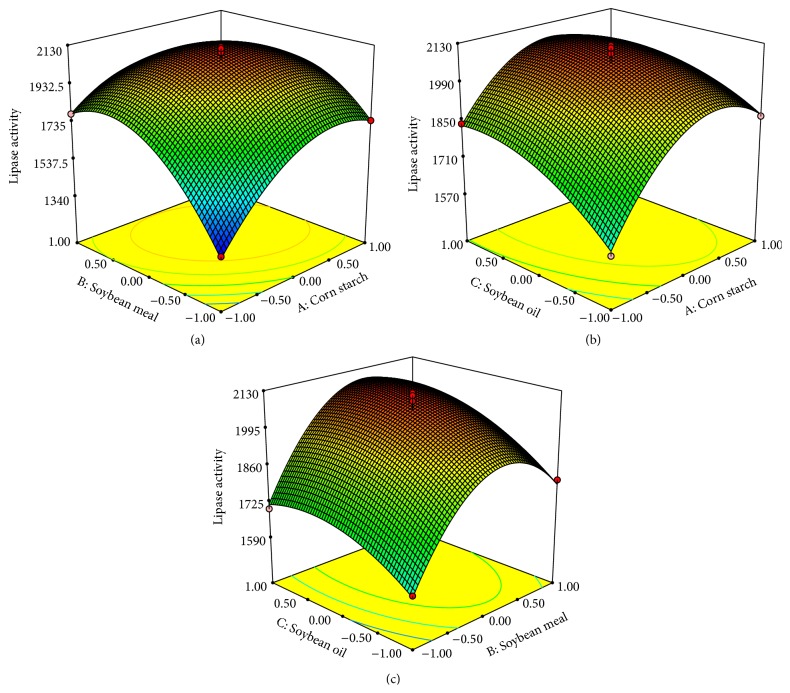
Response surface plot showing the effect of each factor on lipase activity obtained by* A. niger* G783. The medium was modified based on FM2. The values are average of three independent experiments and the error bars represent standard deviation. (a) Corn starch with soybean meal; (b) corn starch with soybean oil; (c) soybean meal with soybean oil.

Earlier studies by Gombert et al. [36] and Dinarvand et al. [37] have optimized C/N ratio in the medium for enzyme production. Comparing to metabolite production (C/N ratio = 300) [38], lower C/N ratio medium is beneficial to enzyme production (C/N ratio <14) [36, 37]. Dinarvand et al. [37] indicated that both organic and inorganic nitrogen sources can improve cell growth and synthesis of enzymes. High productivity of enzymes was obtained under low C/N ratio condition, carbon limitation, and rich nitrogen. The C/N ratio in this BBD study was set to 6.6–8.3 within an appropriate range for enzyme production. As shown in Figure 6(a), the interaction between factors *A* (corn starch) and *B* (soybean meal) was significant, which indicated that C/N ratio (corn starch/soybean meal) was important for lipase production. This result suggested that more attention should be paid to C/N ratio in the optimization of enzyme production. As shown in Figures 6(b) and 6(c), the interactions of *A* and *C* or *B* and *C* were not significant. Although soybean oil not only induces the lipase synthesis but also is used as carbon source [35], our results suggested soybean oil did not significantly affect the C/N ratio.

After derivation of the quadratic equation and calculation (according to Design-Expert 8.0), the concentrations of three factors in the optimum medium for maximum lipase activity production were predicted as 10.5 g/L corn starch, 35.4 g/L soybean meal, and 10.9 g/L soybean oil. Therefore, the optimum fermentation medium (FM3) was predicted as follows (per liter): 25 g glucose, 10.5 g corn starch, 35.4 g soybean meal, 10.9 g soybean oil, 12 g NaNO_3_, 1.5 g NaH_2_PO_4_, 0.2 g K_2_SO_4_, and 5 g CaCO_3_, pH adjusted to 7.2. The maximum lipase activity was predicted at 2,096 U/mL.

### 3.3. Validation Experiments

Validation experiments were carried out in triplicate to confirm the predicted optimal conditions. Initial fermentation medium (FM1), single-factor optimum medium (FM2), and BBD optimum medium (FM3) were investigated and evaluated to validate the predicted optimal conditions (Table 4). The final lipase activity of* A. niger* G783 in FM3 was 2,171 ± 41 U/mL with a slight increase compared to the predicted value. Considering the experimental error, this result was consistent with predicted value, which suggested that the model built by BBD in this study is valuable for optimization of the fermentation medium. Moreover, the lipase activity was significantly improved in FM3 compared to the other two media, especially the initial medium (FM1). This statistical optimization study on* A. niger* fermentation medium is important for industrial lipase production. This study also provided helpful experiences for industrial production strain improvement.

## 4. Conclusions

Medium component significantly affects the revenue in industrial scale fermentation, due to the effect on feedstock cost and product yield. In this study, we successfully demonstrated sequential single-factor experiments and BBD strategy could be used for optimization of fermentation medium for the industrial production strain. Due to its effect on the C/N ratio, corn starch, soybean meal, and soybean oil were selected as factors in BBD design to predict the optimal conditions. Statistical analysis from BBD suggested that the C/N ratio was important for* A. niger* lipase production. Using the predicted condition, the optimum lipase activity of* A. niger* G783 was up to 2,171 ± 41 U/mL, which was 16.4% higher than using the initial medium. This optimal fermentative medium formula could be used for the future upscale lipase production using* A. niger*. This study also provided helpful experiences for industrial production improvement.

## Figures and Tables

**Table 1:** The composition of components and the experimental sequence for the single-factor experiment.

Sequence	Nutrients	Components	Concentration (g/L)
1	Primary carbon sources	Glucose, maltose, sucrose, glycerol, hydrol^a^	15, 20, 25

2	Secondary carbon sources	Corn starch, modified starch, dextrin	8.5, 10, 11.5

3	Nitrogen sources	Soybean meal, yeast extract, beef extract, peptone, sodium nitrate^b^	35, 40, 45

4	Oils	Olive oil, soybean oil, lard oil, peanut oil, sun-flower seed oil	5, 10, 15

5	Inorganic salts	NaH_2_PO_4_, K_2_SO_4_, CaCO_3_	0, 1.5, 2.0, 2.5 (for NaH_2_PO_4_)
			0, 0.15, 0.2, 0.25 (for K_2_SO_4_)
			0, 4, 5, 6 (for CaCO_3_)

^a^Corn starch hydrolyzate (hydrol), a less expensive nutrient source for industrial medium, contains 50–60 g/L glucose and is rich in trace elements. The value of hydrol was 30, 40, and 50 g/L.

^b^40 g/L soybean meal was supplemented in the analysis of sodium nitrate. The concentration of sodium nitrate was set as 8, 10, and 12 g/L, respectively.

**Table 2:** Coded levels and real values (in parentheses) for the BBD and lipase activity achieved after fermentation by *A*. *niger* G783.

Run	*A* Corn starch (g/L)	*B* Soybean meal (g/L)	*C* Soybean oil (g/L)	Lipase activity (U/mL)
1	0 (10)	−1 (30)	1 (15)	1698
2	1 (12)	0 (35)	−1 (5)	1866
3	0 (10)	0 (35)	0 (10)	2017
4	0 (10)	0 (35)	0 (10)	2093
5	−1 (8)	0 (35)	−1 (5)	1575
6	0 (10)	0 (35)	0 (10)	2014
7	0 (10)	−1 (30)	−1 (5)	1600
8	0 (10)	0 (35)	0 (10)	2113
9	0 (10)	0 (35)	0 (10)	2122
10	1 (12)	−1 (30)	0 (10)	1744
11	0 (10)	1 (40)	1 (15)	1970
12	−1 (8)	0 (35)	1 (15)	1837
13	−1 (8)	−1 (30)	0 (10)	1353
14	0 (10)	1 (40)	−1 (5)	1807
15	−1 (8)	1 (40)	0 (10)	1778
16	1 (12)	1 (40)	0 (10)	1754
17	1 (12)	0 (35)	1 (15)	1955

**Table 3:** ANOVA for lipase activity obtained by *A*. *niger* G783.^a^

Source	Degrees of freedom	Sum of squares	Mean square	*F* value	Prob. > *F*	
Model	9	704679.1	78297.7	43.89	<0.0001	Significant
*A*	1	75272.0	75272.0	42.20	0.0003	
*B*	1	104424.5	104424.5	58.54	0.0001	
*C*	1	46818.0	46818.0	26.24	0.0014	
*AB*	1	43056.2	43056.2	24.14	0.0017	
*AC*	1	7482.2	7482.2	4.19	0.0798	
*BC*	1	1056.2	1056.2	0.59	0.4667	
*A* ^2^	1	148065.8	148065.8	83.01	<0.0001	
*B* ^2^	1	217012.0	217012.0	121.66	<0.0001	
*C* ^2^	1	24336.0	24336.0	13.64	0.0077	
Residual	7	12485.8	1783.7			
Lack of fit	3	1475.0	491.7	0.18	0.9057	Insignificant
Error	4	11010.8	2752.7			
Total	16	717164.9				

^a^Coefficient of determination (*R* ^2^) = 0.9828, CV = 2.29%. A model with an *F* value of 43.89 implies that the model is significant, which could occur due to noise. Values of “Prob. > *F*” less than 0.01 indicate that model terms are significant. In this case, *A*, *B*, *C*, *AB*, *A* ^2^, *B* ^2^, and *C* ^2^ are significant model terms. The “Predicted *R* ^2^” of 0.9431 is close to the “Adj. *R*-Squared” of 0.9602, which corrects the *R* ^2^ values for the number of terms and for the sample size in the model. The “adequate precision” value of 22.523 indicates an adequate signal. The lack of fit is insignificant and the model is adequate. This model can be used to navigate the design space.

**Table 4:** Validation experiments of the optimum medium (*n* = 9).

Medium	Lipase activity (U/mL)				
	Batch 1	Batch 2	Batch 3	Mean ± SD	CV (%)
FM1	1863 ± 27	1835 ± 30	1896 ± 62	1865 ± 45	2.18
FM2	2046 ± 34	1996 ± 63	2030 ± 59	2024 ± 52^a^	2.27
FM3	2174 ± 46	2161 ± 54	2177 ± 39	2171 ± 41^b^	1.7

^a,b^Lipase activity in FM2 and FM3 increased by 8.5% and 16.4%, respectively, compared to FM1.
